# A global network for network medicine

**DOI:** 10.1038/s41540-020-00143-9

**Published:** 2020-08-31

**Authors:** Bradley A. Maron, Lucia Altucci, Jean-Luc Balligand, Jan Baumbach, Peter Ferdinandy, Sebastiano Filetti, Paolo Parini, Enrico Petrillo, Edwin K. Silverman, Albert-László Barabási, Joseph Loscalzo, International Network Medicine Consortium, Bradley A. Maron, Lucia Altucci, Jean-Luc Balligand, Jan Baumbach, Peter Ferdinandy, Sebastiano Filetti, Paolo Parini, Enrico Petrillo, Edwin K. Silverman, Albert-László Barabási, Joseph Loscalzo

**Affiliations:** 1 Department of Medicine, Brigham and Women’s Hospital and Harvard Medical School, Boston, MA USA; 2 Department of Precision Medicine, University of Campania Luigi Vanvitelli, Napoli, Italy; 3 Institute of Clinical and Experimental Research (IREC), Faculty of Medicine, Universite´ catholique de Louvain, Brussels, Belgium; 4 Department of Experimental Bioinformatics, Technical University of Munich, Freising, Germany; 5 Department of Pharmacology and Pharmacotherapy, Semmelweis University, Budapest, Hungary; 6 Department of Translational and Precision Medicine, Sapienza University of Rome, Rome, Italy; 7 Department of Laboratory Medicine and Department of Medicine, Karolinska Institutet and Karolinska University Hospital, Stockholm, Sweden; 8 Department of General Internal Medicine and Primary Care, Brigham and Women’s Hospital, Boston, MA USA; 9 Channing Division of Network Medicine, Brigham and Women’s Hospital, Harvard Medical School, Boston, MA USA; 10 Network Science Institute and Department of Physics, Northeastern University, Boston, MA USA; 11 Department of Network and Data Science, Central European University, Budapest, H-1051 Hungary

**Keywords:** Scientific community, Diseases

## Abstract

Network Medicine is now an established scientific discipline, having generated key insights on integrated mechanisms underlying complex human diseases. The next discovery phase will emphasize innovating analytics that optimize networks for advancing precision medicine. Accomplishing this goal will require organizing a scientifically diverse team studying big datasets shared globally.

## Introduction

Network Medicine is a scientific discipline that focuses on the interaction between biological components, such as proteins, microRNAs, or metabolites, to understand molecular pathways that underlie the pathogenesis of diseases. More recently, Network Medicine has expanded to integrate molecular data with phenotypic features as a means by which to clarify mechanisms driving clinical disorders. Since the first publication introducing Network Medicine in 2007^1^, nearly 3300 scientific reports have advanced and refined this discipline, which aims to use scientific “big data” to decipher the role of molecular interactions in the context of health and human disease.

Originating in yeast two-hybrid and tandem affinity purification systems as a means to create protein-protein interaction networks, the arc of Network Medicine has evolved greatly. This evolution includes, for example, demonstrating that clinical disorders previously regarded as dissimilar, in fact, share common protein connectivity features, and informing point-of-care risk stratification tools. Increasingly, Network Medicine is working with ever-larger molecular and phenotypic datasets, and moving into the realm of networks of networks. Both static and dynamic features of these increasingly complex structures will be essential for realizing the field’s full potential in biomedicine. Diversity in Network Medicine analytics has proved gainful^2^, but has given rise to new challenges that must be met in order to optimize the extensive intellectual and data resources available world-wide. Indeed, international efforts in Network Medicine have been somewhat fragmented, hindering opportunity to advance the field maximally. To address this crossroads, the first International Conference on Network Medicine and Big Data assembled in Rome, Italy, in 2018, and determined that an organized scientific approach to Network Medicine, inclusive of international input functioning synergistically, is needed. This goal is particularly timely considering that access to increasingly complex omics platforms will define scientific eras to come, thereby shifting emphasis from acquisition of data to integrative analysis of data as viewed through a network lens.

## Goals and Strategic Overview of Network Medicine

In the last decade, translational research has established multidisciplinary mindsets, thus widening the cultural and scientific opportunities of individual researchers. Despite these gains, translating biological insights to human disease diagnosis and therapeutics remains stagnant. One principal reason for this dilemma may be inherent to conventional, reductionist approaches used for scientific problem solving. Indeed, Network Medicine is antithetical to this time-honored approach, focusing on integrating different layers of information to ascertain the inherent and mechanistically relevant connectivity among biological components. It is in this way that phenotype alignment with pathobiological substrate is often superior, albeit reorganized, as compared to stochastic or probabilistic modeling of simple associations alone^3^.

A distinguishing feature of Network Medicine is the incorporation of all information layers to define pathophenotypes, which is accomplished by expanding data dimensions^4^. Thus, biases commonly ascribed to viewing complex diseases through the prism of a single sentinel event, often a genetic variant, do not exist. In contrast, focusing on protein-protein interactions (as one example) that (i) regulate specific endophenotypes and (ii) facilitate cross-talk between pathways that influence different endophenotypes permits a flexible paradigm inclusive of, but not solely due to, genetic context^5^. The smallest cluster (disease network module) of information within a larger interactome is identifiable, and can be used to discover unique features for disease classification, therapeutic targeting, or drug repurposing^2^.

Network Medicine is well-positioned to advance personalized medicine since the method itself is based on tightly linked functional relationships among epigenomic, transcriptomic, post-transcriptional, proteomic, and metabolomic data. Derivative patterns, including cutting-edge strategies^6^ to individualize datasets tied to patient reticulotypes (i.e., individualized disease modules specific to a patient), offer promising insight into the clinical implications of patient-specific mechanistic biological signatures. Pursuing this strategy will prove important toward developing an optimal health management strategy for aging societies, in particular, for which chronic non-communicable diseases will affect most populations.

## International Network Medicine Consortium (INMC)

The INMC aims to balance hypothesis-driven and data (methodology)-driven approaches to healthcare applications, leveraging the creativity of group members whose expertise is rooted deeply in knowledge of network science, computer science, physics, mathematics, biology, pathophysiology, and clinical medicine. There are three principal scientific goals of the INMC: (1) to explain single cell functions and cell-cell interactions that determine the behavior of the whole organism, and how the whole organism interacts with its environment to maintain homeostasis, which ultimately degenerates into disease states; (2) to apply this information to foster new diagnostic tools, preventive strategies, and therapeutic targets, and to discover new drugs and repurpose approved drugs; and (3) to integrate biological and physiological information contained in networks to discover mechanistically new disease subgroups, enhance patient risk stratification, and develop individualized treatment strategies.

Creating and validating these models requires wide-ranging skill sets, from which analytical pipelines can emerge: (i) data sorting, aggregation, and network construction and analysis; (ii) modeling dynamic interactions; and (ii) translating and confirming data application to biomedical systems. These processes will require critical input from biologists, physicians, computer scientists, physicists, mathematicians, and statisticians. Thus, the creation of the INMC may advance research quality and reproducibility, and lead to a unified understanding of disease mechanisms. An initial focus will be on synchronization and integration in sharing data, creating protocols for evaluating patients so that deep clinical phenotypic data are usable in network analysis, and integrative experimental design. To ensure a stable stream of diverse intellectual input, formal training courses, workshops, and outreach science communication activities will be available for interested stakeholders.

## Model for scientific discovery

The INMC model includes four distinct, but interacting, components: *methods*, *molecular network profiles*, *phenotype network profiles*, *and machine learning and artificial intelligence*. In *methods*, properties of complex networks that can be segmented into sub-networks (n-partite networks) are considered, and the relevance of data determined by network theory informs likelihoods, and, thereby, significance scores. In *molecular network profiles*, genes, proteins, and other molecular data types are used to reveal fundamental biological relationships resulting in a unique network wiring pattern that is the basis for any given disease. Correlation-based, gene regulatory, Bayesian, and protein-protein interaction networks are used; some networks connect diseases to associated factors outside the human cell, and parallel networks of environmental and genetic etiological factors (networks of networks) linked with shared diseases inform the ‘etiome’. Clustering environmental factors in these networks stands to provide critical data on acquired (modifiable) disease triggers, often lacking in conventional reports^7^.

In phenotype network profiles, interactome networks are used to refine disease classification, discover molecular targets for drug repurposing, and establish novel therapeutics^8,9^. Emphasizing disease network modules, which are neighborhoods or subnetworks of components in the interactome that, if disrupted or altered, result in specific pathophenotypes, has already proven to be insightful^10^. Patient-level clinical phenotyping based on networks that include functionally related physiological parameters is also informative, but underutilized^11^. Machine learning focuses on regularities in clinical or molecular data, and is a useful strategy by which to filter network instruments, such as minimal spanning trees or deregulated sub-network identification^12^, in order to identify mechanotypes and novel endophenotypes^13^.

The INMC is an emerging consortium leveraging global intellectual and data resources to create a highly interdisciplinary environment for expanding the discipline of Network Medicine in research, education, and clinical care. By integrating contemporary methodologies, biological platforms, computational technologies, and global intellectual resources, the INMC mission is to refine knowledge of disease pathogenesis and advance drug discovery. Meeting this goal will likely require including an expansive but rational approach to studying biological as well as socioeconomic, nutritional, wellness, and other datasets yielding information on acquired risk for disease. In addition and importantly, for the full adoption of Network Medicine by the biomedical community, novel strategies for training and education will need to be developed. In doing so, challenging, effective, and attainable goals have been set for the next phases of Network Medicine, which promise to revolutionize concepts in medical research for the purpose of optimizing disease treatment and outcome.

## Supplementary information

Supplementary Information

## References

[1] Barabási AL (2007). Network medicine–from obesity to the “diseasome”. N. Engl. J. Med..

[2] Menche. J (2015). Disease networks. Uncovering disease-disease relationships through the incomplete interactome. Science.

[3] Halu A (2019). Exploring the cross-phenotype network region of disease modules reveals concordant and discordant pathways between chronic obstructive pulmonary disease and idiopathic pulmonary fibrosis. Hum. Mol. Genet..

[4] Lee LY, Loscalzo J (2019). Network medicine in pathobiology. Am. J. Pathol..

[5] Maron BJ, Maron MS, Maron BA, Loscalzo J (2019). Moving beyond the sarcomere to explain heterogeneity in hypertrophic cardiomyopathy: JACC Review Topic of the Week. J. Am. Coll. Cardiol..

[6] Liu X, Wang Y, Ji H, Aihara K, Chen L (2016). Personalized characterization of diseases using sample-specific networks. Nucleic Acids Res..

[7] Liu YI, Wise PH, Butte AJ (2009). The “etiome”: identification and clustering of human disease etiological factors. BMC Bioinformatics.

[8] Cheng F (2018). Network-based approach to prediction and population-based validation of in silico drug repurposing. Nat. Commun..

[9] Casas AI (2019). From single drug targets to synergistic network pharmacology in ischemic stroke. Proc. Natl Acad. Sci. USA.

[10] Zhou X, Menche J, Barabási AL, Sharma A (2014). Human symptoms–disease network. Nat. Commun..

[11] Oldham WM (2018). Network analysis to risk stratify patients with exercise intolerance. Circ. Res..

[12] González G (2018). Disease staging and prognosis in smokers using deep learning in chest computed tomography. Am. J. Respir. Crit. Care Med..

[13] Alcaraz N (2017). De novo pathway-based biomarker identification. Nucleic Acid Res..

