# Supplementary material for: A global network for network medicine
Source: NPJ Syst Biol Appl. 2020 Aug 31;6:29. doi: 10.1038/s41540-020-00143-9 (PMC7459285; doi:10.1038/s41540-020-00143-9)
Supplement: Supplementary file 1 — Supplementary Information [file 41540_2020_143_MOESM1_ESM.docx]

**Supplementary Information File: Manuscript NPJSBA-00549R1**

***International Network Medicine Consortium Universities/Institutes and sponsoring representatives**:

Bence Agg, MD^9^, Lucia Altucci, MD, PhD^6^, Eleni Anastasiadou, PhD^7^, Italo F. Angelillo, DDS, MPH^6^, Marcello Arca, MD^7^, Jean-Luc Balligand, MD, PhD^10^, Albert-Laszlo Barabasi, PhD^2,4,5^, Jan Baumbach, PhD^8^, Giuditta Benincasa, MSc^6^, Claudio Borghi, MD^16^, Richard Rosenquist Brandell, PhD^3^, Marina Camera, PhD^14^, Giovambattista Capasso, MD, PhD^13^, Peter J. Castaldi, MD, MSc^1^, Carlo Catalano, MD^7^, Alberico L. Catapano, PhD^14^, Michele Ceccarelli, PhD^13^, Fortunato Ciariello, MD, PhD^6^, Nello Cicenia, JD^12^, Giovanni Cirillo, MD, PhD^6^, Carmine DeAngelis, MD^15^, Sabino DePlacido, MD^15^, David deRidder, MSc^18^, Marcella Devoto, PhD, MSc^7^, Antonella Di Costanzo, PhD^15^, Johanna Diehl, PhD^3^, Beniamino Di Martino, PhD^6^, Giovanni DiMinno, MD^15^, Cosimo Durante, MD, PhD^7^, Vladislav Elgart, PhD^1^, Ingemar Ernberg, PhD^3^, Lorenzo Farina, PhD^7^, Péter Ferdinandy, MD, PhD^9^, Olivier Feron, PhD, MS^10^ , Sebastiano Filetti, MD, PhD^7^, Luigi Formisano, MD, PhD^15^, Giovanni B. Frisoni, MD, PhD^18^, Silvana Galderisi, MD, PhD^6^, Gemma Vilahur Garcia, DVM, PhD^17^, Laurent Gatto, PhD, MSc^10^, Eugenio Gaudio, MD^7^, Antoine Geissbuhler, MD^18^, Mario Giuliano, MD, PhD^15^, Kimberly Glass, PhD^1^, Mauro Iacono, PhD, MSc^6^, Istvan Kovacs, PhD^2^, Volker Lauschke, MBA, PhD^3^, Janne Lehtiö, PhD^3^, Maurizio Lenzerini, PhD^7^, Stefano Leonardi, MD^7^, Jane Leopold, MD^1^, Nisha Limaye, PhD^10^ , Yang-Yu Liu, PhD^1^

, Matthias Löhr, MD, PhD^3^, Joseph Loscalzo, MD, PhD^1^, Bradley A. Maron, MD^1^, Lina Badimon Maestro, PhD^17^, Toni Mancini, PhD^7^, Marianna Maranghi, MD, PhD^7^, Fabrizio Maturo, PhD^6^, Alessandro Mei, PhD^7^, Erik Melén, MD, PhD^3^, Joerg Menche, PhD^2^, Antimo Migliaccio, MD^6^, Umberto, Nanni, PhD^7^, Claudio Napoli, MD, PhD^6^, Ciro Nasti, MD^12^, Vincenzo, Nigro, MD^6^, Paola Paci, PhD^7^, Giuseppe Paolisso, MD, PhD^6^, Michele Papa, MD^6^, Paolo Parini, MD, PhD^3^, Enrico Petrillo, MD^1,11^, Tommaso Pippucci, PhD^16^, Elisabetta Poluzzi, PharmaD, PhD^16^, John Quackenbush, PhD^1^, Guillaume Rey, PhD^18^, Kerry J. Rhoden, PhD^16^, Maria Sabrina Sarto, PhD^7^, Harald Schmidt, MD, PhD, PharmaD^19^

, Marco Seri, MD, PhD^16^, Edwin K. Silverman, MD, PhD^1,4^, Alberto Marchetti-Spaccamela, PhD^7^, Julinda Stefa, PhD^7^, Peter Stenvinkel, MD, PhD^3^, Paolo Tieri, PhD^7^, Elena Tremoli, PhD^14^, Enrico Tronci, PhD^7^, Ruisheng Wang, PhD^1^, George Washko, MD, MS^1^, Guido Valesini, MD^7^, Paola Velardi, PhD^7^, Rosanna Verde, PhD^6^, Antonella Verrienti, PhD^7^, Alessandro Vespignani, PhD^2^, Davide Viggiano, MD, PhD^13^, Andrea Vitaletti, PhD^7^, Ortensio Zecchino^13^

**^12^Fondazione Istituto for Network Medicine, Italy, ^13^Istituto BioGem, Italy, ^14^Universita` degli Studi di Milano, Italy, ^15^Universita` degli Studi di Napoli Federico II, Italy, ^16^Universita´ di Bologna, Italy, ^17^Universitat Autonoma de Barcelona, Spain, ^18^Université de Genève, Switzerland, ^19^Universiteit Maastricht, Netherlands**
